# Antibiotic knowledge, attitudes and behaviours of Albanian health care professionals and patients – a qualitative interview study

**DOI:** 10.1186/s40545-017-0102-1

**Published:** 2017-04-04

**Authors:** Susanne Kaae, Admir Malaj, Iris Hoxha

**Affiliations:** 1 Department of Pharmacy, Section for Social and Clinical Pharmacy, Faculty of Health and Medical Sciences, University of Copenhagen, Universitetsparken 2, 2100 København Ø, Denmark; 2 Faculty of Pharmacy, University of Medicine Tirana, Albania, Fakulteti Farmacise, Rr. Dibres 371, 1000 Tirana, Albania

**Keywords:** Antibiotics, Albania, Interviews, Antibiotic knowledge, Attitudes, Practices, Patients, Pharmacists, Physicians

## Abstract

**Background:**

The inappropriate use of antimicrobials is a problem worldwide. To target future interventions, a thorough understanding of the reasons behind this current behaviour is needed. Within the EU, the culture of antimicrobial use has been intensely studied, but this is not the case in non-EU southeastern European countries, despite the frequent use of (broad-spectrum) antibiotics (ABs) in this region. The aim of this study was to explore AB knowledge, attitudes and behaviours of health care professionals (HCPs) and patients in one southeastern European country, Albania.

**Methods:**

In total, 16 semi-structured interviews were carried out with four groups of interviewees: physicians, community pharmacists, and patients with and without AB prescriptions. Interviews were used to investigate participants’ recent practices with four specific antibiotics for upper respiratory tract infections, along with their typical behaviours, knowledge and attitudes towards the use of antimicrobials. A directed content analysis was applied.

**Results:**

The patients showed little awareness of the differences between viruses and bacteria; however, they often self-diagnosed, which led them to request ABs from pharmacies without a prescription. Pharmacists felt pressured to give in to patients’ demands. All of the participants (including HCP) showed ﻿suboptimal beliefs about illness severity as they all believed that ‘flu complications’, i.e. flu/cold symptoms that persisted after 2–3 days, should be treated with ABs. Physicians usually had no rapid tests to guide them in their practice; however, they were not concerned about this fact. HCPs acknowledged AMR, but only a few of them seemed to consider its risk in their daily practice.

**Conclusions:**

Patients had high levels of trust in and desire for ABs, and HCPs did not often negotiate with patients’ demands. Suggested initiatives to improve the prudent use of ABs in Albania include higher reimbursement for prescribed antibiotics (to reduce illegal sales), academic detailing as well as implementing public awareness campaigns.

**Electronic supplementary material:**

The online version of this article (doi:10.1186/s40545-017-0102-1) contains supplementary material, which is available to authorized users.

## Background

Antimicrobial agents, such as antibiotics (ABs), have dramatically reduced the number of deaths from infectious diseases during the 70 years since their introduction. However, due to the inappropriate use of this type of medicine, many micro-organisms have become resistant to antibiotics [1]. This problem is estimated to cause 25,000 deaths annually in the EU. The costs incurred by drug-resistant infections amount to an estimated €1.5 billion annually due to increases in healthcare expenditures and productivity losses [1].

To combat antimicrobial resistance (AMR), the WHO Regional Office for Europe has initiated several programmes for both EU and non-EU member states. One programme engages in surveillance of drug consumption in the non-EU countries of southeastern Europe. The published results from 13 countries in this geographical area show some indication of inappropriate AB use. Specifically, the findings suggest little consumption of narrow-spectrum penicillin and high consumption of ABs, such as penicillin combinations, third-generation cephalosporins and long-acting macrolides, causing an increased risk of AMR [2].

To support interventions that decrease inappropriate AB consumption, a qualitative multi-country study was collaboratively launched in 2014 by non-EU countries in southeastern Europe, the Health Technology and Pharmaceuticals group of the WHO, the Regional Office for Europe and qualitative researchers from the Section of Social and the Clinical Pharmacy, University of Copenhagen (SSC) [3]. The aim of the qualitative AMR study was to explore AB knowledge, attitudes and behaviours of health care professionals (HCPs – i.e., both physicians and community pharmacists) and patients in each country as a means to target more effective future interventions in the area.

To date, AB knowledge, attitudes and behaviours are factors that have been demonstrated to influence the use of ABs, particularly in western societies. For example, it has been shown that inappropriate prescribing is driven by physicians’ perceptions that patients expect AB prescriptions, fear of disease progression and fear of losing patients to competitors [4–8]. Most HCPs recognize the risk of AMR; however, they are significantly more likely to perceive AMR as a national problem rather than one being affected by their own practice [8, 9]. Contradictory attitudes towards AB prescribing have also been identified among non-western physicians [8]. Furthermore, lack of awareness of appropriate AB use is widespread among patients. One survey found that on average only 48% of EU residents could correctly state that ABs are ineffective against the cold and flu, and participants with low levels of education were more likely to have misconceptions about ABs [10].

In contrast to most western European countries, many eastern European countries are prone to the often illegal sale of over-the-counter (OTC) ABs in community pharmacies; however, some uncertainty exists about the actual extent of the problem. A systematic review published in 2011 found that northeastern European countries, such as Lithuania, Poland and Romania, have a high frequency of OTC sales of ABs (approximately 30%), whereas others have suggested that only approximately 8% of the ABs used in this region are obtained without a prescription [11]. Furthermore, there are indications that OTC sales of ABs have in fact decreased in countries such as Lithuania due to legislation in this area [12]. The review from 2011 also reported that approximately 6% of ABs are sold OTC in some countries in southeastern Europe, i.e., Croatia, Slovenia and Slovakia [13], while other studies have suggested that approximately 50% of ABs are sold OTC in this region [2]. This estimate was supported by a study carried out in Bosnia–Herzegovina in 2010, where 58% of the visited pharmacies illegally sold ABs without a prescription [14].

One southeastern European country that is a part of the qualitative WHO AMR research project is Albania. Albania gained independence in 1912 and is a former communist country with a distinct culture. Albania has a GNP of €10.0 billion (2014), which has constrained the basic education of the public [15], and one-third of its population (2.8 million inhabitants in total) are not covered by a public health insurance policy [16]. Only physicians have the right to prescribe medication and AB should according to law be obtained by prescription only. In recent years, Albania has experienced a rapid increase in the number of community pharmacies, which has challenged the implementation of inspections [17]. The country does not produce or import penicillin V; additionally, specific guidelines for the use of ABs do not exist.

Several of these factors are assumed to contribute to high and inappropriate AB consumption in Albania. This assumption has partly been demonstrated by studies showing that illegal OTC sales of ABs occur in 80% of Albanian pharmacies and that the use of broad-spectrum and penicillin combinations is common practice [18, 19].

It has been argued that interventions addressing physicians’ prescribing practices related to ABs must encompass context-specific actions [20]. Thus, to inform policymakers and other stakeholders of the specific reasons behind the current use of ABs in Albania, the aim of this study was to investigate the AB knowledge, attitudes and behaviours of patients and HCPs in the country.

## Methods

Qualitative semi-structured interviews were selected as the most adequate method to investigate AB knowledge, attitudes and behaviours of patients and HCPs in order to collect detailed accounts of participants’ individual experiences and perceptions regarding AB use in order to understand the culture around this phenomenon in-depth [21].

Four types of interviewees were included, as they were all believed to provide valuable insight into the existing culture of AB use: patients/adults who had used ABs with a prescription, patients/adults who had used/purchased ABs illegally without a prescription, community pharmacists (both admitting to legal and non-legal sales of ABs) and physicians working in the primary care system. Despite the fact that physicians and community pharmacists are the legal and professional gatekeepers to patients’ access to ABs, the influence of patients on for example physicians’ AB prescribing practices has been demonstrated in previous studies [7, 22]. Therefore, it was considered important to incorporate these groups in this study.

The study was restricted to investigate AB use for upper respiratory tract infections (URTIs) for several reasons. First, the majority of ABs prescribed in ambulatory care are used to treat respiratory tract infections, and viruses can cause up to 80% of all URTIs [23, 24], making it an accessible and relevant case for studying inappropriate as well as common uses of ABs. Second, restricting the area of research would allow for more homogenous data, thereby increasing the possibility of identifying stronger patterns. Further, four specific ABs were followed: amoxicillin-clavulanic acid, azithromycin, ciprofloxacin and ceftriaxone. This decision was made to ensure the comparability of the data and also because these specific ABs had been shown to be used inappropriately in the region of southeastern Europe, posing a particular public health risk, as most were broad-spectrum ABs [2].

Seven general research questions were established in relation to investigating and addressing AB knowledge, attitudes and behaviours: the process of diagnosis, how and why a specific AB was selected, where and how ABs were purchased, the patients’ use of ABs, satisfaction with the AB process along with AB knowledge and AB attitudes. The questions were operationalized as open questions in interview guides with some variation between the four groups of interviewees [3]. Hence, pharmacists for example were only interviewed about experiences pertaining to his/her pharmacy practice along with his/her knowledge and attitudes and thus not interviewed about how diagnosis is carried out by physicians or how patients used the purchased AB (please see Additional file 1).

In terms of interview technique, to allow for as detailed accounts as possible, patients were asked to answer the different questions specifically in relation to the most recent occurrence within the last 3 months (to reduce memory bias) in which they had one of the four specific ABs prescribed to them or sold to them at the pharmacy counter for a URTI. Likewise, HCPs were asked to describe two or three consultations during the last week in which they had prescribed or sold a specific AB for a URTI. Hence, this technique of referring to recent specific incidents was applied to generate coherent and detailed narratives of interviewees’ experiences with ABs [25]. For example, a full description of how patients purchased an AB in a pharmacy without a prescription would inevitably also contain aspects of the patients’ knowledge and attitudes towards ABs. Additionally, the narrative would likely include both their and the pharmacists’ behaviour and social interactions [3]. The narratives of the different groups of interviewees could further be compared and used to supplement each other to achieve a more complete picture of how ABs were used in daily life. All of the groups were additionally asked if they had other previous experiences with AB use, and if so, they were asked whether those instances were similar to the specific recent account they had just provided.

The semi-structure of the interviews was thus defined by a pre-developed interview guide with open questions to generate narratives and probing during the interviews according to the achieved answers.

Two interviewers from Albania completed a two-day training course led by two researchers from an established Social Pharmacy research group from the University of Copenhagen. Both of these researchers had substantial experience with conducting semi-structured interviews [26]. The training of the Albanian data collectors included how to conduct the semi-structured interviews in compliance with the specific requirements of the qualitative AMR project.

Since the study was considered exploratory, being the first of its kind in Albania, a convenience yet purposive sampling strategy was used [27]. Specifically, the snowball sampling technique was used in which the Albanian researchers asked people in both their professional and private networks if they knew of patients and HCPs who fit the inclusion criteria. To reduce selection bias, the researchers attempted to ensure heterogeneity with respect to age, gender and education.

The participants were verbally informed about the aim of the project, and all participants provided verbal consent to proceed. The appointments were agreed upon in advance for two reasons: to ethically allow participants to withdraw and to ensure that enrolled patients had begun their AB treatment before being interviewed about their medicine use. The interviews were not recorded due to the culture in Albania in which many people do not feel confident when recorded. Instead, extensive hand-written notes were taken. Most of the interviews were thus conducted by both interviewers, allowing one interviewer to concentrate on taking notes. Two patient interviews revealed that the recent use of ABs was for a URTI affecting the child of the interviewee and not themselves; these interviews were still included, as they were considered to be useful for addressing the study’s research questions.

To ensure feasibility as well as research quality, 16 semi-structured interviews in total were conducted and included in the analysis (four within each interviewee group). Kvale recommends 15 interviews plus/minus 10 interviews when conducting semi-structured interviews in to order to produce validated results [21].

### Analysis

The first step of the analysis involved directed content analyses [28], in which answers from each of the transcripts/notes pertaining to the general research questions were extracted, i.e., relevant answers were deductively identified. In the second step, one participant’s answers were compared with those from other participants within the same group to derive a general understanding of how that group used or thought about ABs. In the third step, the understanding of each interviewee group was compared with the other groups both with regard to knowledge, attitudes and behaviour. Hence, to arrive at a more complete picture of typical AB behaviour, attitudes and knowledge, the developed understanding of each group of interviewees was compared and contrasted with other groups. The identified patterns were then (re-)organized into the initial categories of knowledge, attitudes and behaviour.

Researchers from Albania and a researcher (first author) from the SSC carried out the first step separately, compared their results in a consensus meeting, and then finalized the last steps of the analysis together. This procedure was considered to be optimal for ensuring high validity since the Albanian researchers could identify certain cultural aspects of AB use, whereas the SSC researchers coming from other cultures could identify other aspects.

## Results

The 16 interviews were conducted between the winter of 2014 and the spring of 2015. The overall demographics of the participants are shown in Table 1. Of the 16 participants, 12 were females with patients aged 30 to 59 years (including two mothers of three children between 2 and 6 years of age). The HCPs ranged in age from 27 to 45 years.

**Table 1:** Participant demographics

Patients with prescription				
Age	Gender		Education/work	
30	Male		Secondary education	
Mother of boy of 6 years of age	Female		Higher education	
Mother of children of 2 and 4 years of age	Female		Higher education	
41	Female		Secondary education	
Patients without prescription				
Age		Gender	Education/work	
36		Female	Higher education	
28		Female	Post graduate education	
47		Female	Higher education	
59		Male	Secondary education	
Community pharmacists				
Age/experience		Gender	Location of practice	Size of practice
30–7 years of experience		Female	Urban area	70–100 customers per day
27–4 years of experience		Female	Urban area	100–150 customers per day
29–6 years of experience		Male	Urban area	40–60 customers per day
31–8 years of experience		Female	Urban area	50 customers per day
Physicians				
Age/experience		Gender	Location of practice	Number of consultations
36–9 years of experience		Female	Urban area	10–20 patients per day
45–20 years of experience		Male	Urban area	15–30 patients per day
28–4 years of experience		Female	Rural area	10 patients per day
42–15 years of experience		Female	Urban area	20–25 patients per day

### Knowledge

The patients expressed their belief that ABs fight infection, yet most were unsure exactly how they do so. Only a few patients distinguished between bacterial and viral infections.

The pharmacists stated that ABs should not be used when OTC medicines such as paracetamol and syrups were sufficient. Otherwise, with the exception of one pharmacist who said that ABs should not be used for viral symptoms, the pharmacists provided no clear responses regarding AB recommendations. With regard to the physicians, the majority described how ABs should be used to treat complicated bacterial or viral infections.

Most of the HCPs acknowledged that AB resistance exists, and several had observed some situations where patients returned due to a lack of AB efficiency. However, at the same time, some HCPs appeared to question the seriousness of AB resistance. With the exception of one HCP, the HCPs did not specify any actions in their daily professional practice in which they took this knowledge into consideration.

As no national clinical guidelines on ABs exist in Albania, the HCPs stated that their basis for AB knowledge and AB-related practices came from continuing educational activities, such as materials on the internet, visits from pharmaceutical company representatives, discussions with colleagues, and their formal education.

### Attitudes

A common understanding between all interviewee groups existed that if a patient suffered from flu/cold symptoms for 2–3 days and OTC medicines had not cured the symptoms within this period, then patients needed stronger medication to recover, i.e., ABs. Most of the interviewees described a condition that they referred to as ‘flu complications’.

The patients and HCPs both seemed to have a high level of trust in ABs, particularly amoxicillin-clavulanic acid and azithromycin. The patients appeared to believe that their symptoms could always be cured. In addition, the physicians and pharmacists described the need to give guarantees to patients that the medicines would work and the blame patients placed on HCPs if their conditions were not cured.

A pressure to satisfy patients was thus felt by both physicians and pharmacists. If patients were not satisfied, then they threatened to choose another pharmacy or physician whom they believed could better help them. Both groups of HCPs admitted that this could lead to professional practices that were not always optimal. The pharmacists especially expressed feeling pressured by patients to sell ABs OTC as if they refused patient would then turn to another pharmacy. Hence, situations in which patients requested specific ABs without prescriptions were common. However, the physicians did not express receiving pressure to prescribe ABs in this way because if patients truly wanted ABs, then they would obtain them directly from the pharmacy.

### Behaviour

In addition to the concept of treating ‘flu complications’ with ABs, both the patients and HCPs also described several situations in which ABs were prescribed or sold to prevent further worsening of flu or cold symptoms, e.g., patients who were busy at work.

As described, many patients turned directly to pharmacies to obtain ABs without a prescription. For most interviewees, including physicians, this type of practice seemed to be an accepted behaviour. The patients turned directly to pharmacies for multiple reasons; for example, their symptoms were perceived to be manageable. In these situations, some patients would request a specific AB with which they had previous positive experiences, or they would request a general AB. A pattern of self-diagnosis was thus identified. Patients mainly sought physicians when they had severe symptoms, symptoms that they had not previously experienced or if the situation concerned their children.

In situations where patients requested a general AB, the pharmacists would inquire about the patients’ former experiences with ABs when deciding what action to take. To ensure the effectiveness of the AB, several pharmacists often selected broad-spectrum ABs.

Regarding physician practices, it was observed that diagnostic tests were usually not available at public clinics. The physicians also described that the test results often took several days to receive. Therefore, physicians instead relied on their clinical observations, which they believed to be sufficient in most cases, to make a proper diagnosis. Several physicians, however, expressed that they would like to use rapid tests.

It also seemed to be common practice for physicians to ask patients if they could afford to pay beyond the public reimbursement scheme, which would allow physicians to prescribe a wider range of ABs – including some injectable broad-spectrum ABs.

## Discussion

Several factors were identified that arguably could lead to an increased risk of AMR. The patients showed little awareness of the differences between viruses and bacteria; however, they often self-diagnosed, which led them to request ABs from pharmacies without a prescription. Especially community pharmacists felt pressured to give in to patients’ demands. Pharmacists often chose broad-spectrum ABs to ensure the treatment’s effectiveness. All of the participants (including HCP) showed incorrect beliefs about illness severity as they all appeared to believe that ‘flu complications’, i.e. flu/cold symptoms that persisted after 2–3 days, should be treated with ABs even preventatively. Physicians’ attitudes and practices were also found problematic. Hence, physicians usually had no rapid tests to guide them in their practice; however, they were not concerned about this fact. Further, HCPs acknowledged AMR, but only a few of them seemed to consider its risk in their daily practice.

### Limitations

A relatively small number of people were interviewed within each interviewee group with regard to the typical AB culture thereby challenging the validity of the results. However, due to the specific design of the study, comparisons between the four groups’ were applied. These comparisons allowed researchers to identify relatively consistent patterns across the groups, and hence only consistent patterns are reported in this paper.

As qualitative research in general, this study points to issues of relevance ﻿to the investigated topic and we can therefore not draw conclusions as to whether the results are transferable to all patients and HCPs in the country including the frequency and extent of the observed tendencies. Several of our results have been confirmed by quantitative studies in the country, including the results related to the common practice of selling ABs without a prescription in community pharmacies in Albania and the practice of buying ABs outside the national reimbursement scheme, which underlines the argument that relevant aspects to AB use culture have been identified in this study [18, 19].

The results might be biased as most interviews were conducted in cities, and rural AB practices might differ from these patterns. Additionally, as the participants were recruited through snowballing sampling; some participants were remotely acquainted with the researchers, why they may have exhibited more rational AB behaviour or having higher knowledge about ABs than the general population of patients and HCPs. However, all of the interviewed pharmacists admitted to illegally selling ABs (perhaps due to not recording the interviews), which showed that this type of behaviour was also captured in the sample. The snowball sampling strategy further proved suboptimal since it led to the recruitment of a few patients who did not fit all of the initial inclusion criteria. The challenges of not recording the interviews were the loss of details and a generally higher risk of misinterpreted results due to only having (selective) notes.

Despite these limits, the specific aim of using qualitative methods to explore AB knowledge attitudes and behaviours appears to have been achieved, as several patterns potentially leading to irrational use of AB were identified.

### Reasons behind inappropriate behaviour

The patients often self-diagnosed, and thus the practice of purchasing ABs without a prescription was common. Physicians and especially pharmacists gave in to patient demands despite not always being comfortable doing so. This was a very unfortunate finding since HCPs are the final gatekeepers to the prudent use of ABs.

According to recent literature, pharmacists sell AB illegally OTC (although recognising that this is against the standards set by regulatory authorities) for three overall reasons: commercial interests, feeling pity for/wanting to help the patient or finding it counterproductive to resist patient demand for AB as they could easily then obtain the AB in another pharmacy [29–31]. Pharmacists in Albania seem in particular influenced by the latter i.e. to think that refusing patient to have AB OTC will have no effect why they eventually gave in and sold ABs.

In this study, one of the reasons for patients’ strong desire for ABs (and therefore pressure on pharmacists to dispense) was the belief that ABs could relieve them of bothersome symptoms. This high level of patient trust in ABs has likewise been reported in other countries, and a study conducted in Russia and Lithuania found that prescribing ABs for URTI has become an integral and even common practice for these types of infections [6].

Another strong ‘belief precursor’ found in this study for high AB consumption in Albania was the widely recognized however overestimated condition of ‘flu complications’ in terms of severity and need of AB treatment. Although western physicians also justify prescribing ABs according to symptom severity and duration, ABs were prescribed for ‘flu complications’ of shorter durations and for less serious symptoms than, for example, conditions called ‘toxic’ by British physicians [32]. Patients in western countries have likewise been demonstrated to endure symptoms of RTI for more than 2–3 days before seeking the help of a health care professional [33].

Prescribing ABs as a preventive measure was also observed in this study. This practice has also been reported in the West but likely to a lesser degree and mostly in specific situations. For example, a German study showed a 23% increase in the prescription of ABs on Fridays [34]. This finding was confirmed by a Norwegian study in which physicians tended to prescribe more ABs right before the weekend to help their patients avoid queues at emergency departments over the weekend [35].

### The specific Albanian case

The differences in Albanian HCPs’ quick decisions to prescribe and sell broad-spectrum ABs might be explained by this study’s findings showing that HCPs shared patients’ high level of trust particularly in broad-spectrum ABs and the common idea of ‘flu complications’. This was supported by the results, which showed that some of the HCPs lacked pharmacological knowledge about ABs. Hence, physicians in other countries appear to disagree more often with their patients when prescribing ABs, and they then try to negotiate with patients to find a solution, than what was observed in this study [22].

Another reason for irrational prescribing or dispensing of broad-spectrum AB might pertain to diagnostic uncertainty, which was reported by Albanian pharmacists and likewise reported by physicians in western countries [4]. Whereas many physicians in western countries report relying on delayed prescribing, adhering to guidelines or use a variety of clinical tests as a way to reduce uncertainty [8, 35–37], Albanian pharmacists often choose broad-spectrum ABs as a strategy to reduce uncertainty, i.e., playing it safe. In contrast, Albanian physicians expressed very little diagnostic uncertainty despite the lack of rapid tests, which is an attitude that has also been shown among physicians from other non-western countries [8].

In Sweden it was found that physicians, who used less structured approaches to diagnose the cause of sore throat, were more concerned about differential diagnoses to and complications of the observed condition compared for example to physicians strictly adhering to guidelines or using clinical tests in a structure manner [36, 37]. If this understanding is general and also pertains to Albanian physicians using unstructured diagnostic ﻿approaches, it could explain why Albanian physicians find it necessary often to prescribe AB in these situations which then leads to irrational prescribing.

To target AB behaviours linked to insufficient knowledge and inappropriate attitudes, the underlying culture and social infrastructure have to be taken into account. In Albania, this could include paying attention to the current lack of access to primary care services, as 1/3 of the Albanian population are not entitled to free medical consultations. Reimbursing all or most of the price of regular AB prescriptions could be considered, because at the current time, reimbursements for ABs with a prescription reduce the total price only by approximately 12%, giving patients little financial incentive to seek a prescription from a physician. However, physicians in Lithuania have now suggested the opposite i.e. no reimbursement for ABs at all, as they believe reimbursement leads to higher consumption [12]. Hence, using reimbursement as a regulatory mean to control use of ABs can be challenging.

Other initiatives might include launching public campaigns to raise the public’s awareness about taking ABs in ‘special’ cases, as this has proven successful in other countries [8]. A restructuring of the microbial test system could also be introduced to provide results to physicians earlier [7, 38], although not all of the physicians in this study seemed to believe that rapid tests were necessary. Academic detailing helping physicians to reflect on their own practices could promote an adjustment to this attitude [7, 8], especially as continuing medical education activities are lacking. Currently in Albania, while a continuing education (CE) system for physicians, dentists and pharmacists exists, it is not well structured, and the responsibility falls on the HCP to seek annual credits on CE activities and select the type and field of CE activity in which to participate. Hence, the CE system in Albania is not prioritizing what are the professional needs that HPs have to fulfil. Finally, it is essential that laws regarding the prescribing and dispensing of ABs, are enforced.

## Conclusions

A multitude of reasons for the inappropriate use of antibiotics in Albania was identified. Patients’ exhibited high level of trust in ABs and subsequently requested AB prescriptions or illegally purchasing them over-the-counter, even for flu-like symptoms lasting for 2–3 days. Health care professionals did not appear to negotiate with patients’ demands; pharmacists preferred to play it safe using broad-spectrum antibiotics, and physicians were overly confident in their ability to diagnose infections without rapid tests. Especially pharmacists were found to give in to selling antibiotics because they did not believe that they could change the current system.

Suggested initiatives in the future include introducing higher reimbursement for prescribed antibiotics, academic detailing to avoid misunderstanding of proper antibiotic use and public awareness campaigns.

## Additional file

Additional file 1: Interview-guides. (DOCX 31 kb)

## Abbreviations

ABs: Antibiotics
AMR: Antimicrobial resistance
HCP (including physicians and community pharmacists): Health care professional
OTC: Over-the-counter
SSC: Section of Social and Clinical Pharmacy, University of Copenhagen
URTI: Upper respiratory tract infection
