# Supplementary material for: Antibiotic knowledge, attitudes and behaviours of Albanian health care professionals and patients – a qualitative interview study
Source: J Pharm Policy Pract. 2017 Apr 4;10:13. doi: 10.1186/s40545-017-0102-1 (PMC5379499; doi:10.1186/s40545-017-0102-1)
Supplement: Additional file 1: — Interview-guides. (DOCX 31 kb) [file 40545_2017_102_MOESM1_ESM.docx]

**Additional file 1. Interview-guides**

**Interview-guide – Patients with prescription**

**Introduction**

- Introduce yourself and introduce the study. Tell what the interview will be used for.
- Ask permission to record the interview on tape
- Assure anonymity of the interviewee
- Ask the interviewee to introduce her/him-self, including age, occupation and where they live

**Last face-to-face consultation with physician leading to prescription for an AB for an upper respiratory tract infection**

Process of diagnosis of AB

- When was the last time you got a prescription for an upper respiratory tract infection (should be within the last 3 months)
- What was the situation – what were the symptoms of the patient – for how long?
- Did you have any idea what kind of disease you were suffering from?
- Did you come here by your own initiative or were you encouraged by family, colleagues or friends?
- Which doctor did you seek – why this doctor?
- What did you want from the doctor?
- What did you expect from the doctor?
- How did the consultation go? What happened? What was said – by whom?
- Did the doctor make a diagnosis/ examine you? If yes, how and do you know what the diagnosis was?

Why a specific AB was chosen / Satisfaction with AB prescription process

- Who chose to use an AB? / Were you involved in this decision?
- If the doctor solely made the decision – was any explanation given why to use an AB?
- What AB was chosen? (with regard to product name, form and strength)
- Were you involved in the decision of what specific AB to use?
- If yes, please describe how. If the doctor chose the AB, did he/she explain why to use this specific AB?
- Did the doctor give any instructions on how to use the AB?
- If yes – which ones? Were the instructions given written or orally?
- Were you satisfied with the way the consultation went?
- If yes, why? If no, why not?

Where was the AB purchased/ Satisfaction with AB purchase process

- Where did you purchase the AB prescribed by your GP?
- Why did you choose this place?
- Please describe the circumstances of the purchase? What happened? What was said – by whom?
- Were you involved in which specific AB was purchased? (due to generic substitution or availability of the drug in the etc.) If yes, please describe how.
- Did the pharmacist give any instructions on how to use the AB? If yes – what were they?
- Were the instruction given written or orally?
- Were these instructions similar to the ones provided by the doctor?
- If the pharmacist changed your medicine to another brand - how did you feel about this?
- Were there any challenges with regard to the purchase in terms of price; was the drug in stock in the store, etc.?
- How much medicine did you purchase? Did you purchase all the necessary AB immediately? If not, why so?
- Were you satisfied with the way the purchase went? If yes, why? If no, why not?

Use of AB/ Satisfaction with AB use

- When you came home - how did you use the AB? (How many tablets/ dosages per day for how long?)
- Did this use correspond with the instructions given by the doctor or pharmacist? If yes, why? If no, why not?
- Did you experience any challenges with taking the AB? If yes, which ones?
- Did the AB alleviate or cure your symptoms? If yes, please explain how and how quickly?
- Did the original symptoms reoccur?

**AB use in general**

The last time compared to other times

- Have you had AB prescribed before?
- If yes, approximately how many times? When did it take place?
- What were the symptoms – for how long?
- Did you have any idea what kind of disease you were suffering from?
- Did the consultations you had with your doctor at these times resemble the last time you received a prescription? Did you also receive a prescription at these times?
- Was the diagnosis carried out the same way as the last time you had an AB consultation? If no, describe how it usually took place.
- Was the way the AB was chosen previously similar to the last time you had an AB prescription? If no, please describe how it usually took place.
- Did the doctor give similar instructions about how to use AB compared to the last time you had an AB prescription? If no, please describe how it usually took place.
- Did you suffer previously from any of these symptoms without seeking a GP? If yes, why (at these times) not think it was necessary to seek a GP?
- Did the visits to the pharmacy where you previously purchased your AB prescription resemble the last time you purchased the AB?
- Were you previously involved in which AB was purchased in the same way as the last time you purchased an AB If no, please describe how it usually took place.
- Did the pharmacists previously give you instructions about AB use in the same way as the last time you purchased an AB? If no, please describe how it usually took place.
- The way you took your AB treatment – does this resemble how you took AB before (number of days, compliance with advice of GP or pharmacist, etc.)

Knowledge and attitudes about when to use AB

- Can you explain what AB does in your body?
- From where do you have this knowledge?
- In which situations do you think AB should be used?
- Why do you think that AB should be given in these situations?
- Where do you have this knowledge from? Do you ever discuss these issues with family, friends, colleagues? If so, do you all agree on these matters?
- Are there situations in which you think AB should not be used?
- Which situations are these?
- Why do you think that AB should not be used in these situations? Where do you have this knowledge from?/ Do you ever discuss these issues with family, friends, colleagues? If so, do you all agree on these matters?
- Have you ever seen public campaigns addressing AB use? If yes, what can you remember from these campaigns? Did the campaigns affect you in any way? If yes, in which way? If no, why not?

**Finalizing interview**

- Thank the interviewee for their time.
- Ask if they have any additional comments to what was said during the interview
- Tell the interviewee what will happen to the recordings now

**Interview-guide – Patients without prescription**

**Introduction**

- Introduce yourself and introduce the study. Tell what the interview will be used for.
- Ask permission to record the interview on tape
- Assure anonymity of the interviewee
- Ask the interviewee to introduce her/him-self, including age, occupation and where they live

**Last time when purchased an AB for upper respiratory tract infection without a prescription**

Process of diagnosis of AB/ Where was AB purchased

- When was the last time you purchased an AB for a respiratory tract infection
- What was the situation – what were the symptoms of the patient – for how long?
- Did you have any idea what kind of disease you were suffering from?
- Did you come by your own initiative or were you encouraged by family, colleagues or friends?
- Why did you seek the pharmacy and not a physician?
- What pharmacy did you choose? – why this place?
- What did you want from the pharmacy visit?
- What did you expect from the pharmacy visit?
- How did the consultation go? What happened? What was said – by whom?
- Did the pharmacist make a diagnosis or did you simply order a specific AB?
- If a diagnosis was made - what was your diagnosis? Did the pharmacist examine you? If yes, please describe how?

Why a specific AB was chosen / Satisfaction with AB purchase process

- Which AB was chosen?
- Who chose the AB? (with regard to product name, form and strength)
- If it was the pharmacist – did they give any explanation?
- If it was the patient – why did you choose this specific AB?
- If the pharmacist and the patient collaborated – please describe the conversation.
- Did the pharmacist give any instructions on how to use the AB? If yes – which ones?
- Were the instructions given written or orally?
- Did the pharmacist change your medicine to another brand? If yes, how did you feel about this?
- Were you satisfied with the way the purchase went? If yes, why? If no, why not?
- Were there any challenges with regard to the purchase in terms of price; was the drug in stock in the store, etc.?
- How much medicine did you purchase?
- Did you purchase all the necessary AB immediately? If not, why so?

Use of AB/ Satisfaction with AB use

- When you came home how did you use the AB? (How many tablets/ dosages per day for how long?)
- Did this use correspond the instructions given by the pharmacist? If yes, why? If no, why not?
- Did you experience any challenges in taking the medicine?
- Did the AB alleviate or cure your symptoms? If yes, please explain how and how quickly?

**AB use in general**

The last time compared to other times

- Have you purchased AB medicine before without prescription?
- If yes, approximately how many times? When did it take place?
- What were your symptoms at that time?
- Did you usually purchase AB without prescription in the same or in different pharmacies? Please explain the reasons for choosing either the same or different pharmacies
- Did the consultations you had with the pharmacist previously resemble the last time you purchased an AB?
- Was a diagnosis made during your previous visits where you purchased an AB? If yes or no, how did it take place?
- Who usually decides which AB to use?
- If you simply give an order usually when purchasing AB - which AB did you usually choose? Why did you choose these ones?
- If the pharmacist chose the AB – have they ever given any explanations for these choices?
- Did the pharmacist previously give similar instructions about how to use AB compared to the last time you purchased an AB? If no, please describe how it usually took place?

Knowledge and attitudes about when to use AB

- Can you explain what AB does in your body?
- Where do you have this knowledge from?
- In which situations do you think AB should be used?
- Why do you think that AB should be given in these situations?
- Where do you have this knowledge from?
- Do you ever discuss these issues with family, friends, colleagues? If so, do you all agree on these matters?
- Are there situations in which you think AB should not be used?
- Which situations are these?
- Why do you think that AB should not be used in these situations?
- Where do you have this knowledge from?
- Do you ever discuss such issues with family, friends, colleagues? If so, do you all agree on these matters?
- Have you ever seen public campaign addressing AB use? If yes, what can you remember form these campaigns? Did the campaigns affect you in any way? If yes, in which way? If no, why not?

**Finalizing interview**

- Thank the interviewee for spending their time with you
- Ask if their have any additional comments to what was said during the interview
- Tell the interviewee what will happen to the recordings now

**Interview-guide – Pharmacists who has sold AB (with and) without prescription**

**Introduction**

- Introduce yourself and introduce the study. Tell what the interview will be used for
- Ask permission to record the interview on tape
- Assure anonymity of the interviewee
- Ask the interviewee to introduce her/him-self, including age, education and years of practice
- Ask the interviewee to describe the location of the pharmacy where he/ she works including how many customers they handle per day

**Examples of purchase of AB without prescription for an upper respiratory tract infection within the last week**

- Please provide two typical examples of consultations (preferable typical) where you sold AB with and without prescription for upper respiratory tract infection within the last week – describe the consultations – how did they go?

*(For the following questions – ask them to describe one consultation in detail before describing the next)*

- What was the situation?
- Why did the patient seek you out? - What were the symptoms of the patient and for how long?
- Did the patient tell why he/ she did not seek a physician?
- Who said and did what during the consultation?
- Did you know the patient?
- Did you carry out a diagnosis (could be oral asking questions) or did the patient ask for a specific AB?
- If you carried out a diagnosis, how did you do this?
- What was your rationale for diagnosing this way?
- If you asked questions – which question did you ask?
- If you made a diagnosis – which AB did you select (active substance, strength and form)?
- Why did you choose this specific drug?
- Was the patient involved in the decision making regarding which AB to use?
- If yes, why and how was the patient involved in the decision?
- If the patient simply ordered a special AB – can you remember any details regarding how this was done?
- If the patient ordered a specific AB – why did you comply with this demand?
- Did you pose any questions regarding the choice of AB?
- Did you give any instructions on appropriate AB use?
- If yes, which ones did you provide? What was your rationale for providing this specific instruction?
- Were the instructions given written or orally?
- Did you pose any questions when providing instructions on how to use AB?
- If yes, which ones and why?
- What did you use the answer for?
- Did you conduct generic substitution? If so, what was the patient’s response to this?
- Did you have to give the patient a smaller package size than what corresponds to the entire treatment period?
- If yes, please describe why this was necessary?
- Are you aware of any guidelines regarding dispensing and selling AB?
- Do you always comply with existing guidelines (if they exist!)?
- If yes, why? If no, why not? If no guidelines exist, then how do you make decisions regarding use of AB?

Satisfaction with AB procedures

- In the two cases from last week. Did you feel it was possible to live up to best practice? If yes, why so? It not, why so? (lack of time? fulfilling patients expectations? lack of drug in store, etc.?)
- Do you ever feel forced to either sell an AB in a way that you do not feel is optimal?
- If yes, can you please provide some examples?
- Why isn’t it possible to always to carry out what is optimal?
- Do you ever feel pressure from different stakeholders (the owner of the pharmacy, patients, regulators, others?) to sell ABs in a specific way?
- If yes, can you please provide some examples? In what ways do you feel pressured? Do you sometimes have to give in to the pressure?

**AB purchase in general**

Examples as compared to usual practice

- Do the two examples where you recently sold AB for an upper respiratory tract infection resemble similar situations in which you sell these ABs?
- In what ways were the two cases typical or untypical with regard to your usual way of diagnosing/ complying patient orders, choosing specific AB, involvement of patient and instructions on AB use?
- Do you sell the specific ABs purchased in the two examples for other indications without prescription than the ones described above?
- What are the most typical indications for which you usually sell (these) AB without prescription?
- If no guidelines exist - how do you decide the best way to treat different infections?

General knowledge and attitude

- For which indications do you think AB should be purchased without a prescription?
- Why do you think that AB should be purchased for these indications?
- Are there indications for which you think AB should not be sold?
- Which indications/ situations are these?
- Why do you think that AB should not be purchased in these situations?
- Do you think that AB resistance is a problem in your country?
- If yes, why? What do you think is the cause of the problem? If not, why so?
- Where do you have your basic knowledge from about AB diagnosis, choice of specific AB as well as how to use specific AB?
- How do you keep yourself up to date in these matters?
- Have you ever seen public campaigns addressing AB use? If yes, what can you remember from these campaigns? Did the campaigns affect you in any way? If yes, in which way? If no, why not?
- When was the last time you received a visit by a sales representative?
- Do you think these visits are helpful in obtaining good purchase practices?

**Finalizing interview**

- Thank the interviewee for spending their time with you
- Ask if their have any additional comments to what was said during the interview
- Tell the interviewee what will happen to the recordings now

**Interview-guide – Physicians**

**Introduction**

- Introduce yourself and introduce the study. Tell what the interview will be used for.
- Ask permission to record the interview on tape
- Assure anonymity of the interviewee
- Ask the interviewee to introduce her/him-self, including age, education and years of practice
- Ask the interviewee to tell about the location of their practice, if they share it with other physicians and how many patients he/she sees per day

**Examples of AB prescription of AB for an upper respiratory tract infection within last week**

Process of diagnosis leading to prescription on AB

- Approximately how often did you prescribe AB for upper respiratory tract infection within the last week?
- Can you describe two typical consultations in which you prescribed AB for an upper respiratory tract infection within the last week?
- In each of these consultations – what happened? Who said and did what?

*(For the following questions – ask them to describe one consultation in detail before describing the next)*

- In each of the two situations - why had the patient come to see you? What were their symptoms and for how long?
- Did you know the patient?
- Did you make the diagnosis in each of the two situations or was it decided already?
- If you carried out a diagnosis – how did you do that?
- What was your rationale for making the diagnosis in this way?
- Did you follow guidelines (if they exist!) when diagnosing? If yes, which ones? If no, why not?

Why a specific AB was chosen/ Knowledge and attitudes

- In each of the two situations why did you choose an AB?
- Did you follow guidelines (if they exist) when choosing to use an AB? If yes, which one? If not, why not?
- In each of the two situations, why did you select the specific AB (with regard to both active substance, form and strength)?
- What was your rational for making this decision?
- Did you follow guidelines (if they exist) when choosing the drug? If yes, which ones? If no, why not?
- Did you include the patient in the specific decision making?
- If yes, why and in what way? If no, why not?
- Did you feel that the patient expected an AB?
- If yes, did you feel pressured to prescribe?
- Did you give any instructions to the patient on how to use the drug? If yes, which ones?
- Were the instructions given written or orally? What was your rationale for giving these particular instructions?
- Did you follow any guidelines (if they exist) regarding this task? If not, why so?
- If no guidelines exist - how did you decide how to diagnose, how to treat and which AB instructions to give?

Satisfaction with AB procedures

- In these two cases from last week - did you feel it was possible to live up to best practice?
- If yes, how? It not, why so (lack of time? fulfilling patients expectations? lack of rapid tests?)
- Do you ever feel forced to either diagnose or prescribe an AB in another way than what you feel is optimal?
- If yes, can you please provide some examples?
- Why isn’t it possible to always carry out what is optimal?
- Do you ever feel pressure from different stakeholders (patients, regulators, peer physicians? others?) to diagnose or prescribe in a special way?
- If yes, can you please provide some examples? In which way do you feel pressured? Do you sometimes have to give in to the pressure?

**AB use in diagnosing and prescribing in general**

Examples as compared to usual practice

- Are these two situations you have been describing typical or untypical to situations where you usually prescribe AB for upper respiratory tract infections?
- In which ways were the two cases typical or untypical with regard to your usual way of diagnosing, choosing specific AB, involvement of patient and instructions on AB use regarding respiratory tract infections?
- The specific ABs prescribed in the two consultations do you prescribe these ABs for other indications than upper respiratory tract infection?

General knowledge and attitude

- For which type of indications do you think ABs should be prescribed?
- Why do you think that AB should be prescribed for these indications?
- Are there indications for which you think AB should not be prescribed?
- Which indications are those?
- Why do you think that AB should not be used in these situations?
- Do you think that AB resistance is a problem in your country?
- If yes, why? What do you think is the cause of the problem? If not, why so?
- From where do you have your basic knowledge about AB diagnosis, choice of specific AB as well as how to use specific AB?
- How do you keep yourself updated in these matters?
- Have you ever seen public campaign addressing AB use? If yes, what can you remember form these campaigns? Did the campaigns affect you in any way? If yes, in which way? If no, why not?
- When did you the last time receive sales representatives by the medicine industry?
- Are these visits helpful in obtaining good prescribing practices?

**Finalizing interview**

- Thank the interviewee for spending their time with you
- Ask if their have any additional comments to what was said during the interview
- Tell the interviewee what will happen to the recordings now
